# Supplementary material for: Efficient Charge Separation and Transport in Fullerene-CuPcOC8 Donor–Acceptor Nanorod Enhancing Photocatalytic Hydrogen Generation
Source: Nanomaterials (Basel). 2024 Jan 24;14(3):256. doi: 10.3390/nano14030256 (PMC10856716; doi:10.3390/nano14030256)
Supplement: Supplementary file 1 [file nanomaterials-14-00256-s001.zip › nanomaterials-2804845-supplementary.pdf]

Supporting Information

Efficient Charge Separation and Transport in Fullerene-CuPcOC$_8$ Donor-Acceptor Nanorod Enhancing Photocatalytic Hydrogen Generation

Zihui Hua [1,2], Bo Wu [1,2*], Yuhe Zhang [1], Chong Wang [1,2], Tianyang Dong [1], Yupeng Song [2,3], Ying Jiang [1,2] and Chunru Wang [1,2*]

Dedicated to Professor Chunli Bai on the occasion of his 70th birthday

[1] Beijing National Laboratory for Molecular Sciences, Key Laboratory of Molecular Nanostructure and Nanotechnology, Institute of Chemistry, Chinese Academy of Sciences, Beijing 100190, China; zhhuaa@iccas.ac.cn

[2] University of Chinese Academy of Sciences, Beijing 100049, China

[3] Key Laboratory of Photochemical Conversion and Optoelectronic Materials and CityU-CAS Joint Laboratory of Functional Materials and Devices, Technical Institute of Physics and Chemistry, Chinese Academy of Sciences, Beijing, 100190 China

* Correspondence: zkywubo@iccas.ac.cn (B.W.); crwang@iccas.ac.cn (C.W.)

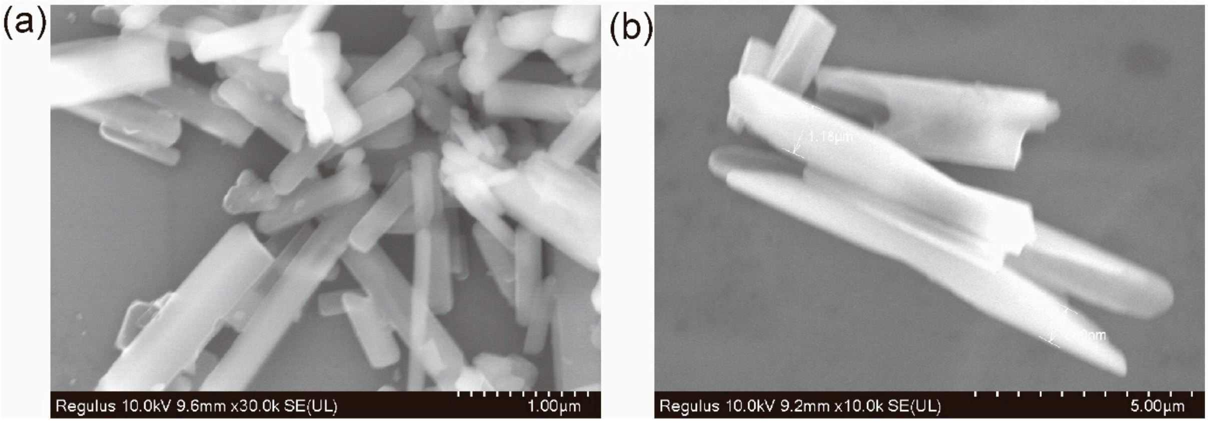

Figure S1. SEM for (a) C_{60} and (b) CuPcOC$_8$.

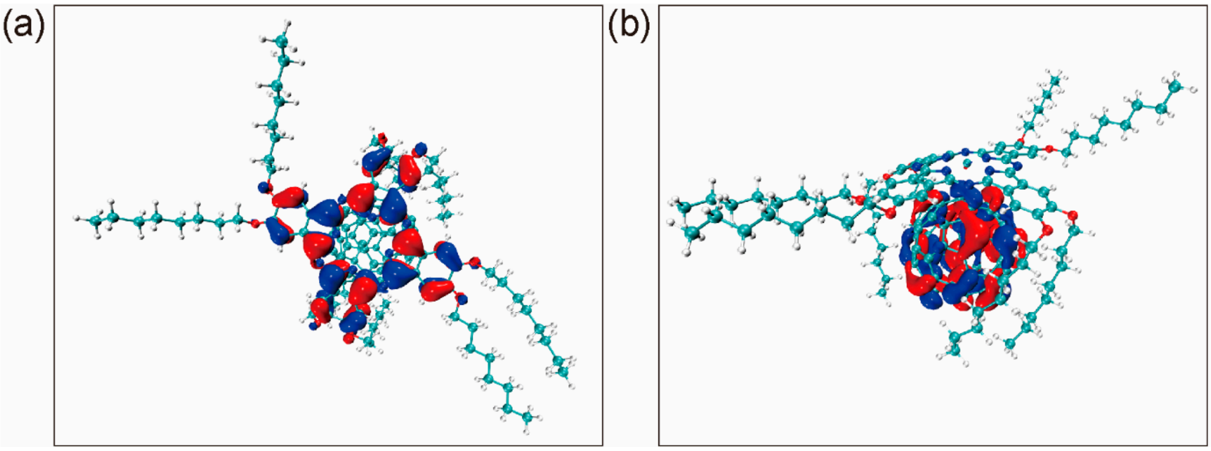

Figure S2. (a) LUMO and (b) HOMO of C_{60}-CuPcOC$_8$.

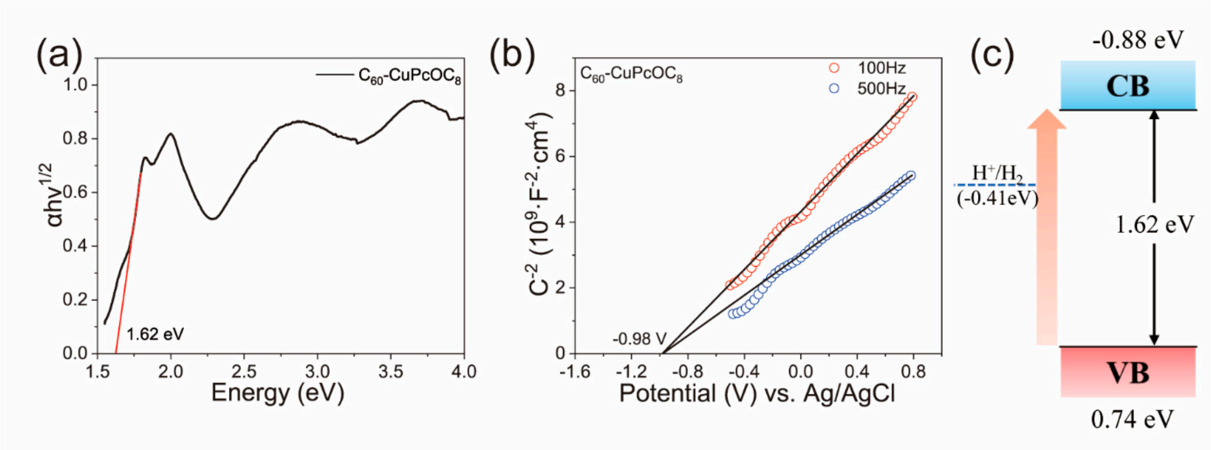

Figure S3. (a) Tauc-plots of C_{60}-CuPcOC$_8$, (b) Mott-Schottky plots of C_{60}-CuPcOC$_8$, (c) schematic representation of the redox potential of C_{60}-CuPcOC$_8$.

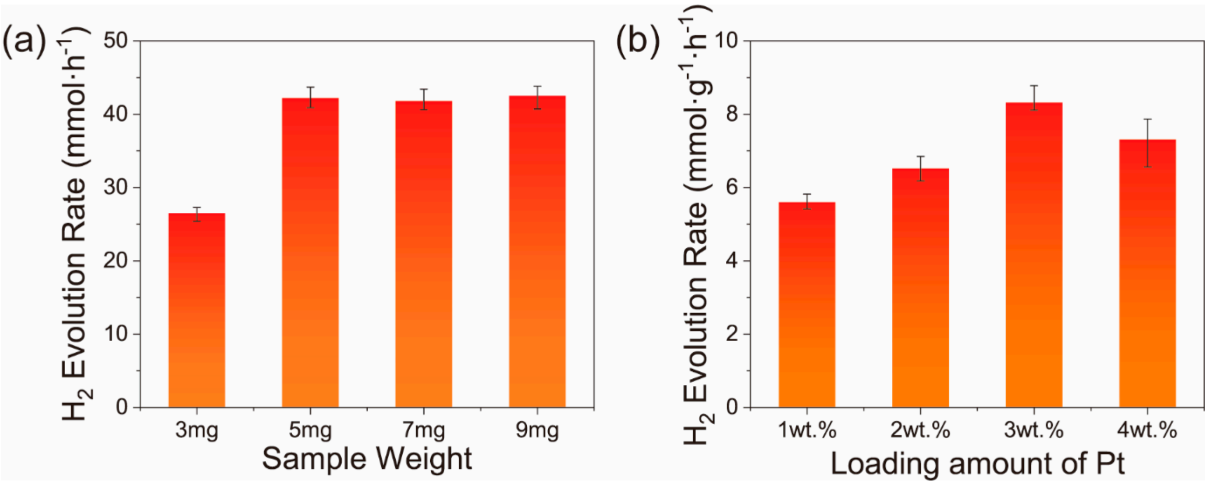

Figure S4. Photocatalytic H_2 evolution rate of C_{60}-CuPcOC$_8$ with varied (a) weight and

(b) loading amount of Pt under full-spectrum light.

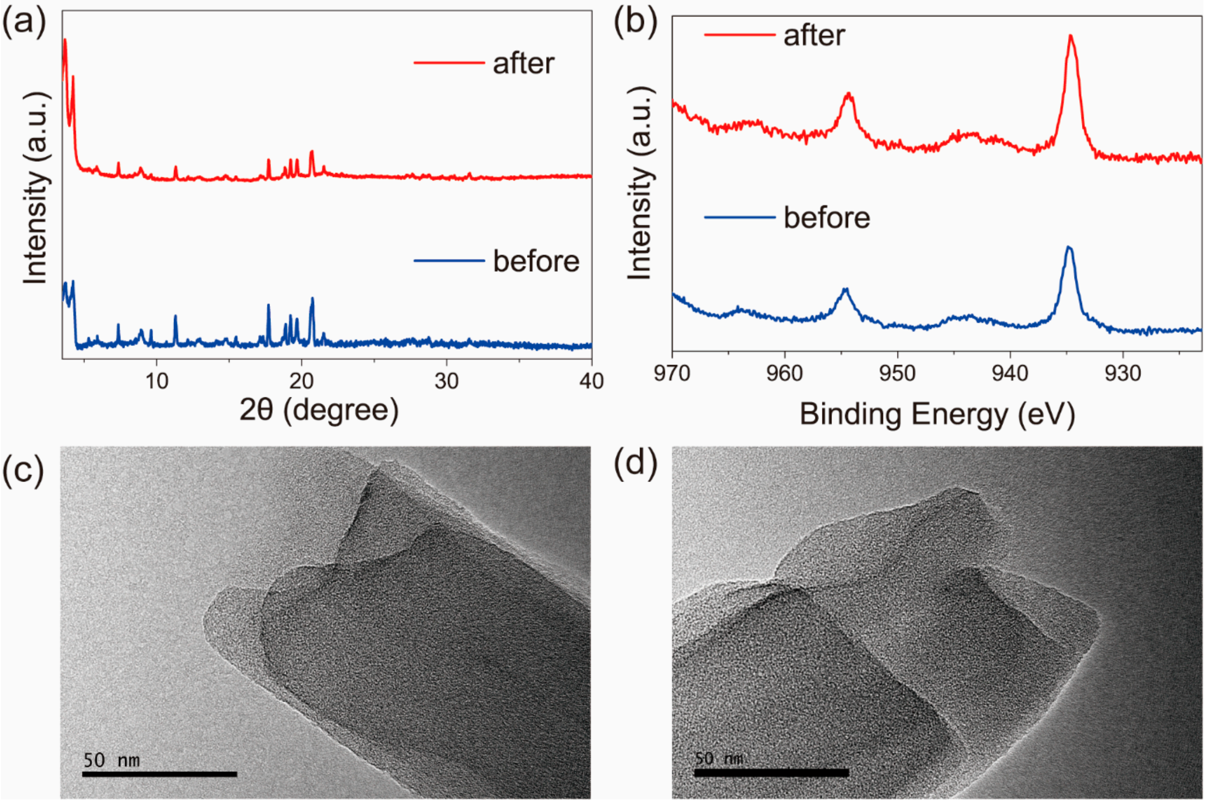

Figure S5 (a) PXRD of C_{60}-$CuPcOC_8$ before and after photocatalytic hydrogen generation; (b) Cu elemental XPS of C_{60}-$CuPcOC_8$ before and after photocatalytic hydrogen generation; HRTEM of C_{60}-$CuPcOC_8$ (c) before and (d) after photocatalytic hydrogen generation.

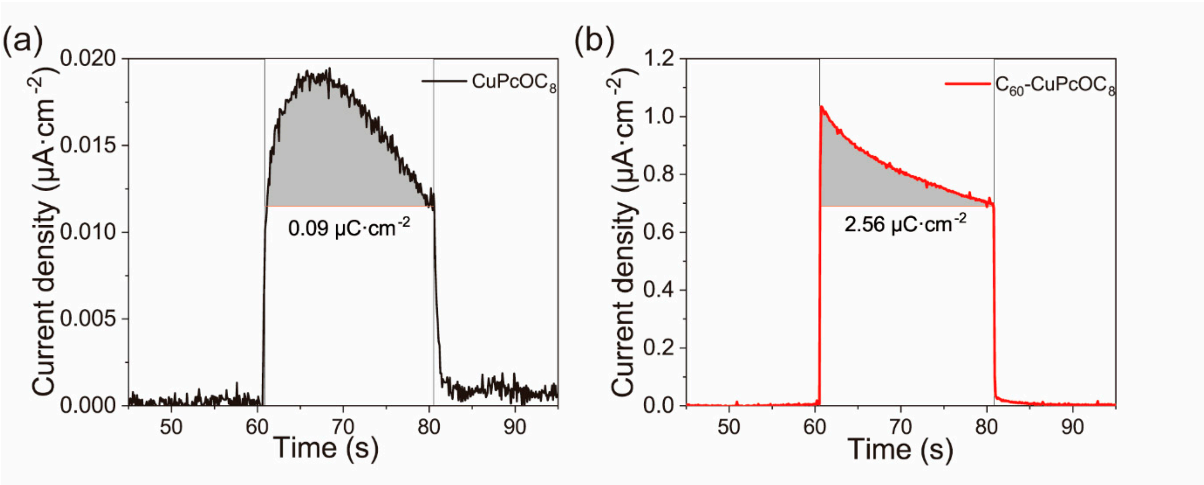

Figure S6. Surface charge density of (a) $CuPcOC_8$ and (b) C_{60}-$CuPcOC_8$.

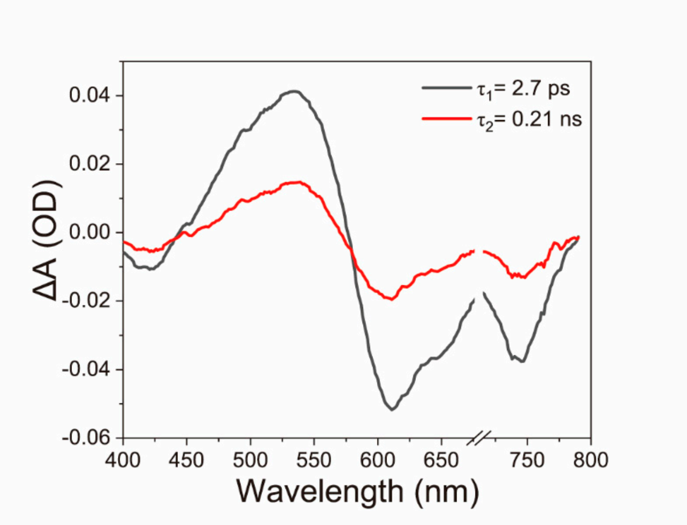

Figure S7. Evolution associated spectra (EAS) of $CuPcOC_8$ at 380 nm (1×10^{-5} M, aqueous dispersion).
